# How Can We Get Close to Zero? The Potential Contribution of Biomedical Prevention and the Investment Framework towards an Effective Response to HIV

**DOI:** 10.1371/journal.pone.0111956

**Published:** 2014-11-05

**Authors:** John Stover, Timothy B. Hallett, Zunyou Wu, Mitchell Warren, Chaitra Gopalappa, Carel Pretorius, Peter D. Ghys, Julio Montaner, Bernhard Schwartländer, the New Prevention Technology Study Group

**Affiliations:** 1 Futures Institute, Glastonbury, Connecticut, United States of America; 2 Imperial College London, London, United Kingdom; 3 China Centers for Disease Control, Beijing, China; 4 AIDS Vaccine Advocacy AVAC, New York, New York, United States of America; 5 British Columbia Centre for Excellence in HIV/AIDS, Vancouver, Canada; 6 United Nations Joint Programme on HIV/AIDS (UNAIDS), Geneva, Switzerland; 7 World Health Organization (WHO), Beijing, China; Alberta Provincial Laboratory for Public Health/ University of Alberta, Canada

## Abstract

**Background:**

In 2011 an Investment Framework was proposed that described how the scale-up of key HIV interventions could dramatically reduce new HIV infections and deaths in low and middle income countries by 2015. This framework included ambitious coverage goals for prevention and treatment services resulting in a reduction of new HIV infections by more than half. However, it also estimated a leveling in the number of new infections at about 1 million annually after 2015.

**Methods:**

We modeled how the response to AIDS can be further expanded by scaling up antiretroviral treatment (ART) within the framework provided by the 2013 WHO treatment guidelines. We further explored the potential contributions of new prevention technologies: ‘Test and Treat’, pre-exposure prophylaxis and an HIV vaccine.

**Findings:**

Immediate aggressive scale up of existing approaches including the 2013 WHO guidelines could reduce new infections by 80%. A ‘Test and Treat’ approach could further reduce new infections. This could be further enhanced by a future highly effective pre-exposure prophylaxis and an HIV vaccine, so that a combination of all four approaches could reduce new infections to as low as 80,000 per year by 2050 and annual AIDS deaths to 260,000.

**Interpretation:**

In a set of ambitious scenarios, we find that immediate implementation of the 2013 WHO antiretroviral therapy guidelines could reduce new HIV infections by 80%. Further reductions may be achieved by moving to a ‘Test and Treat’ approach, and eventually by adding a highly effective pre-exposure prophylaxis and an HIV vaccine, if they become available.

## Introduction

In 2011 a new Investment Framework for HIV/AIDS was proposed to guide efforts in the coming years towards the rational use of resources to confront the AIDS epidemic [1]. The Investment Framework called for all low- and middle-income countries to focus on a set of Basic Programs of proven effectiveness: i) prevention of mother-to-child transmission (PMTCT), ii) condom promotion and distribution, iii) programs for key populations (in particular sex workers and clients, men who have sex with men, people who inject drugs), iv) treatment, care and support for those living with HIV, v) voluntary medical male circumcision (in countries with low prevalence of male circumcision and high HIV prevalence), and vi) targeted behavior change programs. The Framework also called for country-specific decisions about the implementation of additional programs, called Critical Enablers, including program enablers (management, procurement, distribution, research, and program communications) and social enablers (such as outreach for HIV testing and counseling, advocacy, mass communications, community mobilization, and activities aimed at stigma reduction and the realization of human rights). The Framework also recognizes that programs should align with broad development objectives and, therefore, also support key development areas where synergies will be high. These include social protection for children, education, legal reform, gender equality, reduction of gender-based violence, poverty reduction, health system strengthening, community systems, and workplace programs.

The annual resources needed to implement this approach in 139 low- and middle-income countries were expected to increase to about US$ 24 billion by 2015 and decline thereafter due to increased efficiencies and a progressive reduction in disease burden due to decreasing morbidity and significantly reduced numbers of new infections. The full implementation of the Investment Framework would be expected to avert at least 12·2 million new infections and 7·4 million AIDS deaths by 2020, and thus provide a cost-effective means to achieve the goals of the 2011 United Nations General Assembly Political Declaration on HIV/AIDS^2^ for 2015, such as reducing sexual transmission by 50%, reducing transmission among those who inject drugs by 50%, and virtual elimination of mother-to-child transmission.

Substantial progress has taken place since the investment framework was first put forward in 2011. Key relevant areas include the potentially large secondary preventive benefit of treatment on HIV transmission, and the individual level benefit associated with earlier initiation of therapy. These finding are now reflected in the 2013 WHO treatment guidelines which recommends ART for all HIV+ adults with CD4 counts below 500 cells/mm^3^, all HIV+ children below the age of 5, all HIV+ pregnant women and all HIV+ adults with active TB disease, co-infected with HBV with severe liver disease, or in serodiscordant partnerships[2]. Furthermore, there has been a renewed interest in the potential impact of ‘Test and Treat’, and eventually other new highly effective prevention technologies including pre-exposure prophylaxis (PrEP), and an HIV vaccine. Therefore, we undertook the present analyses to examine the potential impact of these strategies on the HIV epidemic through 2050.

Previous modeling work has investigated the potential impact of scaling up ART coverage [3]–[5], pre-exposure prophylaxis (PrEP) [6]–[7] and potential HIV vaccines [8]–[9]. The modeling results differ somewhat across different models but conclusions about magnitude and timing of impact have generally been similar. The Goals model, used for this analysis, produces results that are comparable to those produced by other models [10]. This study builds on this previous work by examining the impact of achieving high coverage of all existing HIV prevention interventions and three new approaches on the HIV epidemic in all low and middle income countries.

## Methods

Similar to the work undertaken for the 2011 Investment Framework, we used the Goals model [11], part of the Spectrum software package, to model the potential impact of different interventions in 24 countries accounting for 85% of new infections in low- and middle-income countries as classified by the World Bank in 2012 (Table 1). Goals is an HIV transmission model that simulates the spread of HIV by modeling sexual contacts and needle sharing behavior. It is a compartment model that divides the population into different categories based on behaviors. Its structure is similar to other compartment models although the specific population groups included differ across models. Its structure is different from microsimulation models that create discrete populations of individuals and characterize each individual with randomly assigned characteristics based on population data. Both types of model can be used to investigate the impact of HIV interventions. The results are expected to be broadly similar when comparable inputs are used.

**Table 1:** Countries for Which Detailed Modeling was Done by Epidemic characteristics.

Hyperendemic, low circumcision	Generalized	Concentrated
Botswana	Cameroon	Brazil
Lesotho	Côte d’Ivoire	Cambodia
Mozambique	Ethiopia	China
South Africa	Kenya	India
Swaziland	Malawi	Indonesia
Zambia	Nigeria	Mexico
Zimbabwe	Tanzania	Russian Federation
	Uganda	Thailand
		Ukraine
		Viet Nam

**Figure 1:**
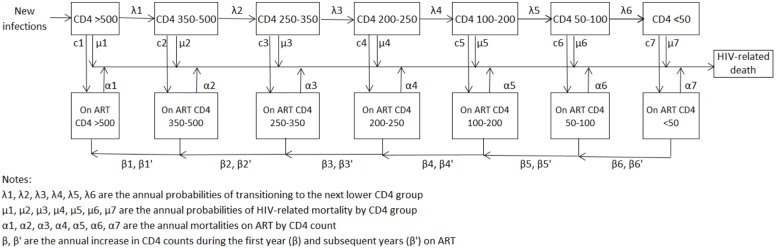
Progression of HIV-infected adults from new infection to HIV-related death.

The adult population aged 15–49 is divided into 11 main risk groups, six for men and five for women: those who are not sexually active, those in stable partnerships with a single partner in the last year, those with multiple partners in the last year, female sex workers and their male clients, people who inject drugs and, men who have sex with men. The annual probability of a susceptible person becoming infected in a risk group is modeled as a function of the probability of encountering an infected partner, the number of acts per partner, the number of different partners per year, the proportion of acts protected by condoms, the prevalence of other sexually transmitted infections, the stage of infection of the infected partner, whether the male partner is circumcised, whether the infected partner is using antiretroviral therapy (ART) and whether the susceptible partners is using PrEP or has been vaccinated against HIV. Most sexual contacts take place between males and females in the same risk group but some proportion of contacts are between higher risk individuals and their low risk partners. Biomedical interventions (ART, male circumcision, condom use, STI treatment, PrEP, vaccination) act directly on the probability of transmission per act and the effect sizes are based on randomized control trials and other scientific studies. Behavioral change interventions influence key behaviors (proportion of acts covered by condom use, number of partners, age at first sex, needle sharing behavior) and the effects are based on studies of behavior change interventions. Those who are newly infected are tracked over time by CD4 count, age and ART status and are subject to non-AIDS mortality as well as CD4-dependent risk of HIV-related mortality (Figure 1). Mother-to child transmission is determined based on fertility rates, HIV prevalence among women of reproductive age and the coverage of prophylaxis to prevent transmission. HIV-infected children are tracked by CD4 count, CD4 percent and ART status. Additional information on the Goals model and its application to these countries is described in File S1.

To model the impact of existing interventions we used the same coverage targets as in the Investment Framework (Table 2 and File S1 “Assumptions about the Existing Investment Framework”). While some countries have already met or exceeded some of these levels the assumption that all countries would achieve them by the target dates is very ambitious. We have used them in these scenarios in order to maintain comparability with the earlier Investment Framework calculations. In addition to the interventions in the Investment Framework, we now also model the impact of the implementation of the latest WHO guidelines regarding initiation of ART with high coverage targets (Table 2 and File S1 “Assumptions about an enhanced Investment Framework”).

**Table 2:** Coverage Targets for the Investment Framework (IF) and Investment Framework Enhanced (IFE).

	InvestmentFramework 2015	InvestmentFrameworkEnhanced 2015	InvestmentFrameworkEnhanced 2020
PMTCT	90%	90%	90%
Condoms (discordant couples)	60%	60%	60%
Condoms: medium risk populations in countries withhyper-endemics and generalized epidemics	60%	60%	60%
Condoms: medium risk in populations in countrieswith concentrated and low level epidemic	20%	50%	50%
Condoms (high risk populations)	50%	50%	50%
Sex work	60%	60%	60%
MSM	60%	60%	60%
IDU outreach	60%	60%	60%
IDU needle and syringe exchange	60%	60%	60%
IDU drug substitution*	40%	40%	40%
**ART among adults**			
ART CD4<200 cells/µl	80%	80%	90%
ART CD4 200–250 cells/µl	70%	70%	90%
ART CD4 250–350 cells/µl	45%	70%	80%
ART CD4 350–500 cells/µl	5%	30%	80%
Pregnant women >500 cells/µl	0%	80% (2017)	80%
Sero-discordant couples and adults co-infected withTB or HBV >500 cells/µl	0%	30%	80%
**ART among children**			
2010 guidelines: <2 years old, 3–5 years old withCD4 count <750 cells/µl, 5+ years old with CD4count <350 cells/µl	80%		
2013 guidelines: <5 years old, 5+ years old withCD4 count <500 cells/µl		80%	80%

*in concentrated epidemics.

Several new approaches and technologies could contribute to future prevention efforts. First, we consider the provision of ART to all people living with HIV regardless of CD4 counts or clinical criteria (Test and Treat), which is currently recommended in North America and elsewhere [12]–[14] and has been shown to reduce infectiousness dramatically [15]. (While the term ‘Test and Treat’ has been used in very different ways by different experts and communities, for this paper we use the term to mean the provision of ART to people living with HIV who are not eligible under the new 2013 WHO treatment guidelines, with CD4>500 cells//µl and not part of a defined population group.) Additionally, we consider several hypothetical scenarios where we explored the potential additional impact of a future highly effective Pre-Exposure Prophylaxis (PrEP) and, a preventive HIV vaccine. PrEP is available today both as oral pills and as vaginal gel but trials have shown widely varying efficacy rates, which have been attributed to suboptimal adherence [16]–[21]. Here we use the term PrEP to refer to all forms of ARV-based prophylaxis including the oral PrEP and vaginal gel formulations as well as new forms that may become available in the future, including long-lasting vaginal rings and injectable forms that are expected to improve adherence, and therefore overall effectiveness. We have assumed that wide scale implementation would wait until these new forms are available. Effective HIV vaccines are still under development and may not be available until around 2030, assuming current technological challenges will be overcome [22]–[24]. A cure may become an important component in the future response to AIDS. However, since this is still in early stages of development, we did not include this option in the current work. The assumptions for each prevention technology are shown in Table 3. For each technology we modeled a ‘low’ or pessimistic scenario using conservative assumptions about coverage for each technology and the introduction date for a vaccine, and a ‘high’ or optimistic scenario with higher coverage and earlier availability for vaccines. Full descriptions of the new technologies and the research behind the assumptions are given in File S1 (under Assumptions about New Prevention Technologies).

**Table 3:** Scenario Definitions for New Prevention Technologies.

Technology	Population Groups	Year of FirstAvailability	Year TargetCoverage isAchieved	TargetCoverageLow/HighScenarios	Effectiveness	Cost
Test and Treat	All other HIV+populationwithCD4counts >500 cells/µl	2014	2025	40%/60%	80% (60%,96%for sensitivityanalysis)	$515 perpatient peryear in 2013falling to$445 by 2027)
Pre-ExposureProphylaxis	MSM	2013	2025	20%/60%	Before 2018:44%; After2018: 70%/90%	$95 perperson peryear
	Female sexworkers	2018	2025	10%/25%		$95/p/y
	Discordantcouples	2020	2025	10%/30%		$95/p/y
	Adolescents in hyper-endemics	2018	2025	0%/30%		$95/p/y
Vaccine	Adultpopulation ingeneralizedepidemics	2025 (high)2030 (low)	2032 (high) 2035 (low)	40%/70%	60% (low)80% (high)	Low income:$20/$12 Middleincome: $55/$35*
	High- riskpopulation inconcentratedepidemics	2025 (high)2030 (low)	2032 (high) 2035 (low)	30%/60%	60% (low)80% (high)	Low income:$20/$12 Middleincome: $55/$35*

Note: For each new technology we modelled two scenarios: a ‘low’ or pessimistic scenario with conservative assumptions about coverage and, for vaccines, availability dates and a ‘high’ or optimistic scenario with higher coverage rates and earlier vaccine introduction.

*Vaccine costs are assumed to be different by income level. For low income countries the cost is assumed to be $20 per regimen at introduction dropping to $12 after 10 years. For middle income countries the cost is assumed to be $55 per regimen at introduction dropping to $35 after 10 years.

The impact of these new technologies was illustrated by modeling several different scenarios:

- Investment Framework (IF): 2011 Investment Framework targets.
- Investment Framework Enhanced (IFE): same as IF but with 2013 WHO Treatment Guidelines implemented in all countries.
- Test and Treat (T&T): expands on IFE by providing ART to 40%/60% of HIV+ adults with CD4 counts >500 cells/µl.
- Pre-exposure prophylaxis (PrEP): expands IFE by providing PrEP to MSM, female sex workers, discordant couples in all countries and adolescents in hyper-endemic countries.
- HIV Vaccine (Vaccine): expands IFE by providing an effective HIV vaccine to 40%/70% of all adults in generalized epidemics and to 30%/60% of high risk populations in concentrated epidemics.
- Combined: combines IFE with the Test and Treat, PrEP and Vaccine scenarios.

## Results

**Figure 2:**
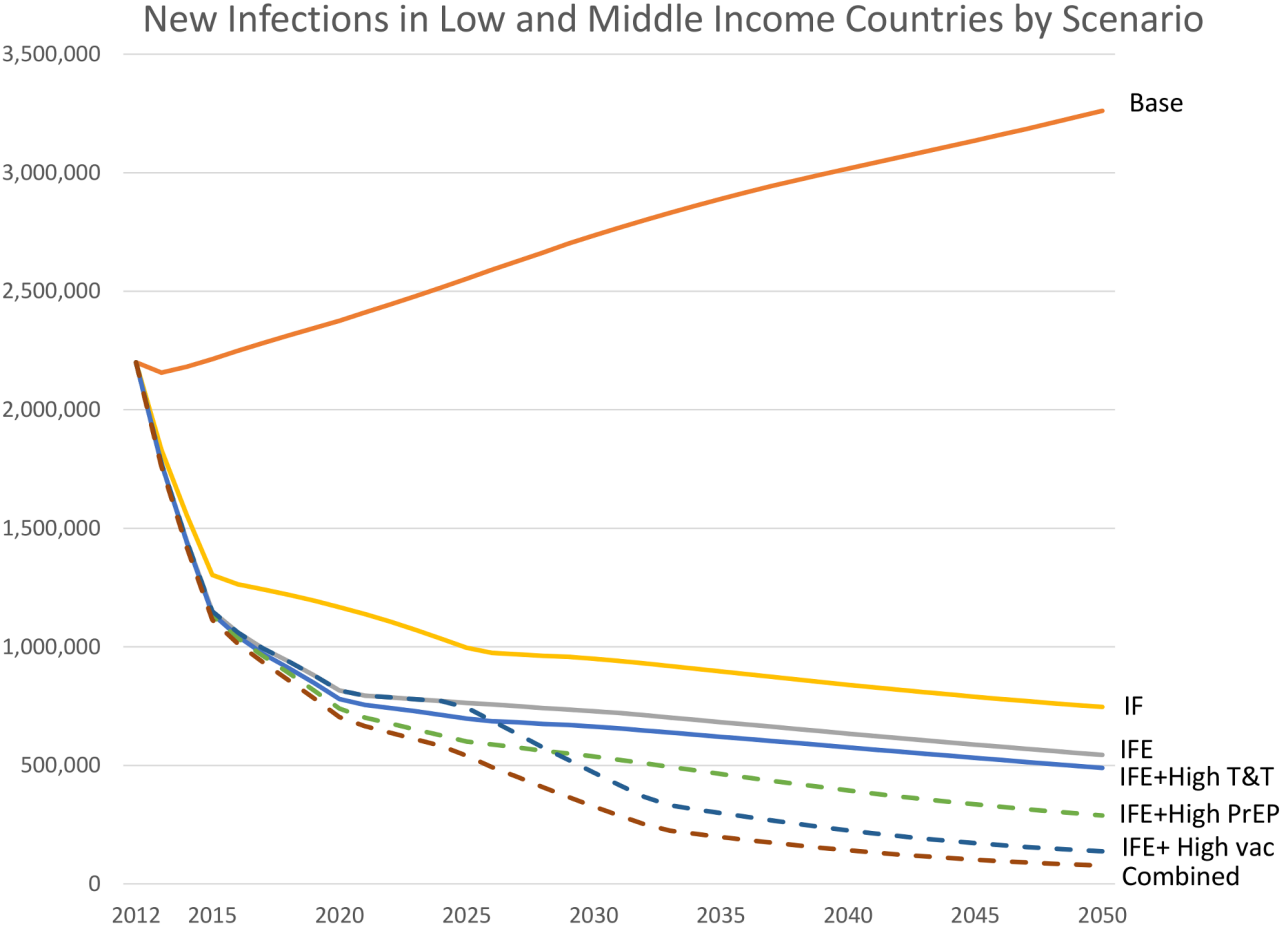
New HIV Infections in Low- and Middle-Income Countries by Scenario. Key: Base = base projection with constant coverage of existing interventions and no new technologies. IF = achievement of Investment Framework 2015 targets scenario. IFE = IF plus adoption of WHO 2013 treatment guidelines**.** IFE + T&T = IF Enhanced plus impact of high Test and Treat scenario. IFE + High PrEP = IF Enhanced plus impact of high PrEP scenario. IFE+ High vac = IF Enhanced plus impact of HIV high vaccine scenario**.** Combined = combination of Investment Framework Enhanced and all three new technologies. Note: solid lines denote scenarios using existing technologies, dashed lines denote scenarios using technologies under development.

If no progress is made towards meeting the coverage targets of the Investment Framework and current coverage of prevention and treatment interventions remains constant at 2012 levels we can expect the total annual number of new HIV infections in low- and middle-income countries to increase slowly mainly due to population growth to about 3·3 million by 2050 (Figure 2). An expansion of prevention and treatment programs to achieve the targets of the original Investment Framework (the IF scenario) could reduce the annual number of new infections in low- and middle-income countries by 77% by 2050 compared to 2011, but about 750,000 people would still be newly infected each year (Figure 2). More comprehensive ART coverage in line with the new WHO 2013 treatment guidelines (the IFE scenario) could reduce annual new infections to 540,000 (or 83%) by 2050.

Next, we consider the impact of adding each new approach by itself to the Investment Framework Enhanced scenario. Adding a ‘Test and Treat’ strategy might reduce new infections in 2050 by an additional 6–10% compared to the IFE scenario to 490,000–510,000 in the low and high coverage scenarios. Implementing a future highly effective PrEP intervention could reduce overall new HIV infections by 16–47% to 290,000 to 450,000 in the low and high coverage scenarios. Finally, under the hypothetical scenario where an effective preventive vaccine becomes available for widespread use by 2025 to 2030 new infections in 2050 would be reduced by 37–75% compared to the Investment Framework Enhanced to 140,000–340,000.

The combination of the IFE with ‘Test and Treat’, plus a future highly effective PrEP and a vaccine, could reduce new HIV infections in 2050 by 86% in the high scenario and 50% in the low scenario resulting in just 80,000 (50,000–110,000) in the high scenario to 270,000 (200,000–380,000) annual new infections in the high scenario. (The ranges represent the sensitivity analysis on the magnitude of reduction in infectiousness due to ART with 96% as the high estimate which corresponds to the HPTN 052 trial [15] and a low value of 60% assuming lower adherence in the general population.) The most optimistic scenario combining all present and future prevention technologies could reduce the cumulative number of new infections from 2013 to 2050 by 96%.

**Figure 3:**
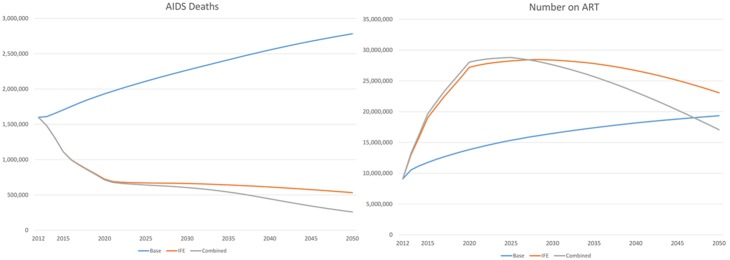
HIV-Related Deaths and Number Receiving ART in Low- and Middle-Income Countries by Scenario. Key: Base = base projection with constant coverage of existing interventions and no new technologies. IFE = IF plus continued increases in ART coverage under WHO 2013 treatment guidelines. NPT = IFE + High T&T, PrEP, Vaccine = combination of IFE and high impact for all three technologies combined.

The number of AIDS-related deaths will also be reduced dramatically by these scale-up scenarios (Figure 3a) decreasing from 1·7 million in 2011 to 260,000 to 430,000 by 2050. This decline results from the rapid scale up in ART and fewer new infections. The number of people receiving ART in the IFE scenario would increase to 19 million by 2015, peak at 28 million by 2028 and decline to about 23 million by 2050 (Figure 3b). It is interesting to note that while by 2050 all three scenarios have similar numbers of people receiving ART, the latter two ART scale-up scenarios would avert about 50–60 million AIDS-related deaths. By 2050, about 1/3 of HIV+ adults would be on ART under the Base Scenario (constant coverage) whereas 82–96% of HIV+ adults would be on ART under the Investment two ART scale-up scenarios (IFE and Combined NPT).

**Figure 4:**
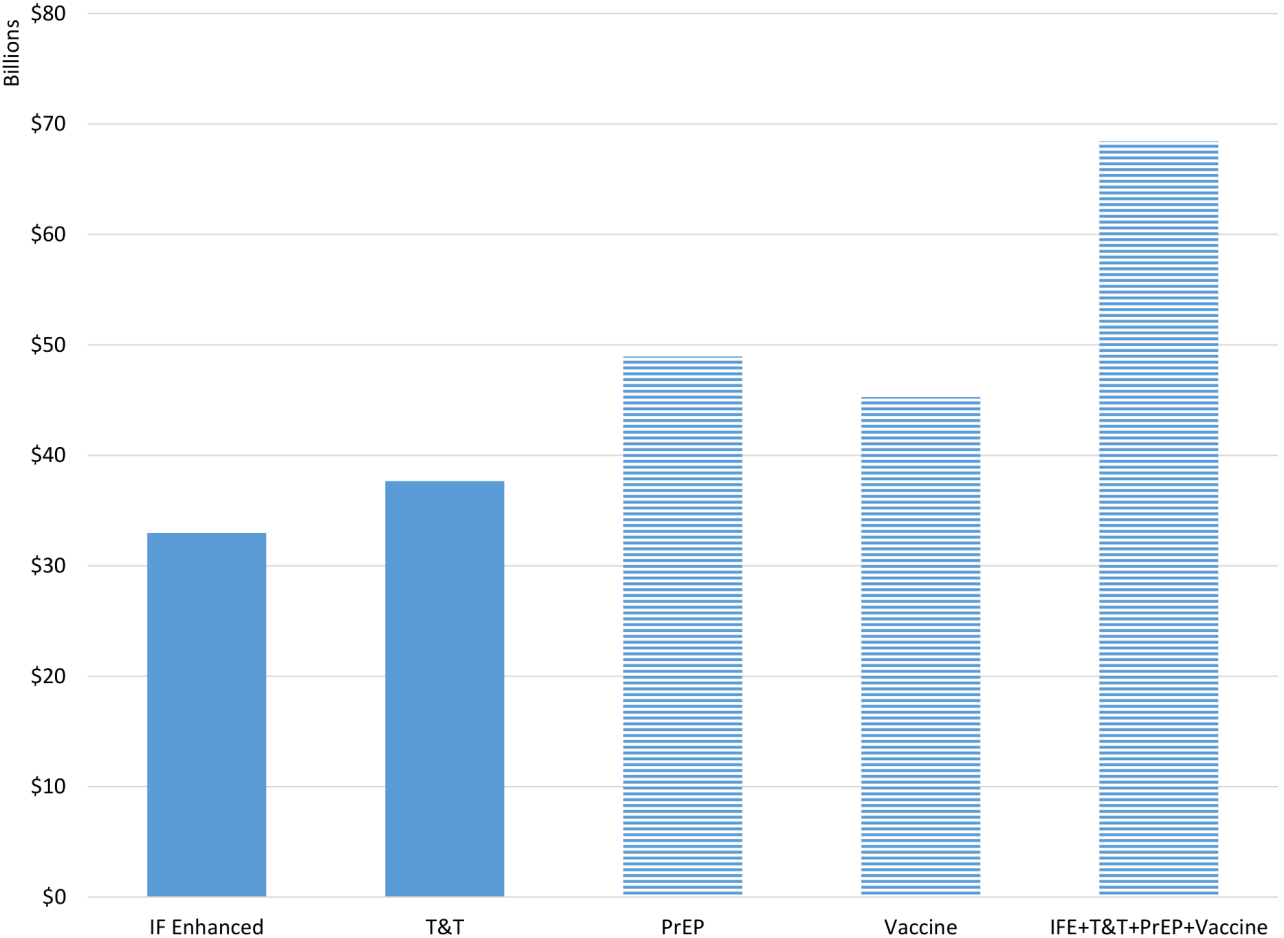
Cumulative Additional Resources Required by Scenario from 2011–2050 in Billions of US$ Discounted at 3% Compared to the Investment Framework 2015. Key: IF Enhanced = IF plus adoption of WHO 2013 treatment guidelines. T&T = IF Enhanced plus high Test and Treat. PrEP = IF Enhanced plus high PrEP scenario. Vaccine = IF Enhanced plus high vaccine scenario. IFE+T&T+PrEP+Vaccine = IFE plus high scenarios for T&T plus PrEP plus HIV vaccine. Note: solid bars denote scenarios using existing technologies, striped bars denote scenarios using technologies under development.

Each of these approaches implies different costs. The total costs are estimated as in the Investment Framework with the additional costs for these new approaches added assuming costs of $515 per person on ART, $95 per person receiving PrEP and $12–$35 per person vaccinated. (Details are provided in File S1.) Figure 4 compares the additional costs of each scenario to the ‘Investment Framework’ scenario. Costs are cumulative from 2011 to 2050 and discounted to 2011 at 3% per year. The Combined scenario requires the most additional resources and the Investment Framework Enhanced scenario the least. In all scenarios large savings are produced by averting new infections and, thus, future treatment needs. These savings partially offset the costs of treating a larger proportion of those eligible for treatment.

The costs per Quality Adjusted Life Year (QALY) gained are shown in Table 4. The costs range from $390 per QALY gained for the Investment Framework Enhanced compared to the original Investment Framework to $11,000 for the low vaccine scenario compared to the Investment Framework Enhanced. The cost per infection averted (not shown) is $6800 when comparing the Investment Framework Enhanced to the Investment Framework and $7300 when comparing the combined scenario with all three new prevention technologies to the Investment Framework Enhanced scenario.

**Table 4:** Impact of Scaling-up Current and New Prevention Approaches.

Scenario	New HIVInfections in2050 (Millions)	Reduction in NewInfections in 2050	Reduction in TotalNew Infections2011–2050	Incremental Cost perQALY Saved 2011–2050
Base – Constant coverage	3·3			
		**Compared to Base**	**Compared to Base**	**Compared to Base**
Investment Framework (IF)	1·1	77% (Compared tobase case)	61% Compared tobase case)	$160 Compared to Base)
		**Compared to IF**	**Compared to IF**	**Compared to IF**
Investment FrameworkEnhanced (IFE)	0·5	27%	20%	$390
		**Compared to IFE**	**Compared to IFE**	**Compared to IFE**
IFE + Low Test and Treat	0·50	6%	3%	$980
IFE + High Test and Treat	0·49	10%	6%	$1,060
IFE + Low PrEP	0·45	16%	6%	$3,500
IFE + High PrEP	0·29	47%	19%	$3,800
IFE + Low Vaccine	0·34	37%	9%	$11,280
IFE + High Vaccine	0·14	75%	26%	$1,160
IFE + Low T&T, PrEP, Vac	0·27	50%	17%	$4,160
IFE + High T&T, PrEP, Vac	0·08	86%	37%	$2,400
IFE + High T&T, PrEP, Vacat 96% ART Effectiveness	0·05	88%	35%	$2,740
IFE + High T&T, PrEP, Vacat 60% ART Effectiveness	0·11	85%	40%	$1,890
IFE + Low T&T, PrEP, Vacat 96% ART Effectiveness	0·20	52%	16%	$4,560
IFE + Low T&T, PrEP, Vacat 60% ART Effectiveness	0·38	49%	17%	$3,490

Our results are highly sensitive to the assumptions we have made about the levels of coverage that could be achieved for each new technology and the effectiveness that would be possible in programs. We have thus included a sensitivity analysis that may give a broad indication on how the different assumptions may play out in terms of impact on new HIV infections. As mentioned above the estimated number of new infections in 2050 varies from 80,000 in the most optimistic scenario to 270,000 in the low or pessimistic scenario.

## Discussion

The present analysis explored the magnitude of incidence and mortality reductions that might be possible by 2050 as a result of the implementation of the 2013 WHO treatment guidelines, and newer available and hypothetical biomedical interventions. We find that expanding treatment coverage expeditiously based on the 2013 WHO treatment guidelines is a critical foundational step of an effective strategy. While such an approach would be challenging to implement, if it can be successfully achieved it would result in marked decreases in progression to AIDS and premature deaths among HIV infected persons and reductions in new infections. Additional resources would be required to achieve this result but at US$ 390 per QALY gained this investment would be very cost-effective.

Embracing ‘Test and Treat’ could further contribute to a decrease in new HIV infections and to a lesser extent a decrease in progression to AIDS and premature deaths among HIV infected persons. The addition of ‘Test and Treat’ to the Investment Framework Enhanced may avert an additional 6–10% of new infections by 2050. Similarly, the addition of a future highly effective PrEP in a targeted fashion could prevent an additional 22% and a hypothetical highly effective preventive vaccine could prevent an additional 30%.

The impact could be much lower if high quality in program is not attained, underlying the sensitivity of our estimates to key assumptions about what can be achieved and which reinforces the need to main quality in programs.

Although we have seen important success in preventing new infections over the past decade, the lack of greater progress partially reflects limited growth in funding and human resources constraints but also indicates the difficulty of achieving higher coverage of current interventions. The leveling off in international resources [25] since 2008 is of concern in this regard. Successful implementation of current approaches (such as outreach to high risk populations, condom use, male circumcision) requires demand for services among the populations at risk, political will to implement these programs, and continuing efforts to reach key populations year after year.

Expanding treatment in line with the 2013 WHO guidelines and Treatment as Prevention requires a substantial expansion in the number of people on ART while at the same time maintaining quality for both new and existing patients. It also requires a large expansion in testing to identify people living with HIV earlier in their infection and it requires willingness on the part of those with high CD4 counts to start treatment before symptoms of HIV infection appear. These factors have previously been identified as major drivers of both the cost and impact of expanded treatment programs, although the costs of outreach are not captured here [26]. If it is not possible to identify such a high proportion of the HIV-infected population or achieve high adherence to treatment and PrEP then these results cannot be achieved. Efforts need to be focused both on expanding coverage of the intervention as well as on ensuring high quality and adherence. Of note, over time the actual number of individuals who need ART converge for all strategies evaluated here; however, the more aggressive the ART roll out, the lower the number of AIDS related deaths.

Our study is limited by only considering a small selection of the possible strategies available. The modeling approach we have used makes a number of simplifying assumptions about patterns of risk behavior and the impact of interventions, but the model has previously been shown to produce results in qualitative agreement with other models making alternative sets of assumptions [27]–[28] which provides reassurance that these simplifications will not interfere materially with the conclusions drawn. Overall, our aim is to provide a useful estimate of the magnitude of impact that may be expected for a set of scenarios. The relationship between the chosen intervention scenarios and how real programs will evolve in the coming years is the most important source of uncertainity.

Our study shows that additional investment now would have a dramatic and lasting impact on the epidemic over a short period of time and on a sustained basis. Under ambitious assumptions, this could be expected to prevent 63 million AIDS related deaths, and 88 million new HIV infections in low and middle income countries by 2050 compared to no expansion of prevention and treatment beyond today’s coverage.

## Supporting Information

File S1 Contains further information on the assumptions for the Investment Framework and Investment Framework Enhanced, more detailed descriptions of Test and Treat, PrEP and HIV vaccines and the sources we used in defining their characteristics, and a full description of the Goals model, including the model equations. **Figure S1**. Risk Structure of Goals. **Figure S2**. Characteristics determining transmission of HIV.
(DOCX)
