# Supplementary material for: How Can We Get Close to Zero? The Potential Contribution of Biomedical Prevention and the Investment Framework towards an Effective Response to HIV
Source: PLoS One. 2014 Nov 5;9(11):e111956. doi: 10.1371/journal.pone.0111956 (PMC4221192; doi:10.1371/journal.pone.0111956)
Supplement: File S1 — Contains further information on the assumptions for the Investment Framework and Investment Framework Enhanced, more detailed descriptions of Test and Treat, PrEP and HIV vaccines and the sources we used in defining their characteristics, and a full description of the Goals model, including the model equations. Figure S1. Risk Structure of Goals. Figure S2. Characteristics determining transmission of HIV. (DOCX) [file pone.0111956.s001.docx]

**Web Appendix**

**How can we get close to Zero? The potential contribution of biomedical prevention and the investment framework towards an effective response to HIV and AIDS**

**Assumptions about the Existing Investment Framework**

The Investment Framework published in 2011 envisions reaching at least 60% coverage of most Basic Programs by 2015 and higher coverage for prevention of mother-to-child transmission (PMTCT) programs (90%) and for individuals eligible for ART under the 2010 WHO treatment guidelines (80% of those with CD4 counts <200 cells/μl, 70% for 200-250 cells/μl, 45% for 250-350 cells/μl and 5% for 350-500 cells/μl) as shown in Table 2. These are minimum targets that are assumed to be reached by all countries not already at or above these levels. For this study, we used the 2011 investment framework as a baseline, projecting that coverage of the key interventions would remain at their target levels through 2050.

We assumed that ART may under optimal circumstances reduce the rate of HIV-transmission by 80% due to viral suppression among a vast majority of those on ART and applied this value for all levels of CD4 counts and in all scenarios assuming delivery through high quality programs. We further conducted sensitivity analyses considering ART effectiveness on HIV transmission of 60% and 96% protection, representing lower and higher levels of adherence and viral suppression, respectively.

**Assumptions about an enhanced Investment Framework**

Next, we projected the long-term impact by assuming that treatment coverage would be scaled up more rapidly in response to the new 2013 WHO guidelines. The ‘Investment Framework Enhanced (IFE)’ scenario assumes 80% coverage for CD4 counts <250, 70% coverage for 250-350 and 30% coverage for 350-500 by 2015, and a continued increase in coverage after 2015 reaching 90% coverage for those with CD4 counts <250 cells/μl, 80% for those with CD4 counts 250-350 cells/μl, and 80% for those with 350-500 cells/ μl by 2020. In addition ART coverage would increase between 2013 and 2017 and reach 80% of all pregnant women who would remain on treatment after initiation, irrespective of their CD4 count, and by 2020 ART coverage would reach 80% of HIV-positive partners in serodiscordant couples with CD4>500 cells/μl plus those co-infected with tuberculosis or Hepatitis B virus. Coverage for all groups remains constant at target levels after 2020.

TABLE 2 ABOUT HERE

**Assumptions about New Prevention Technologies**

Several new approaches and technologies that are not included in the 2011 Investment Framework could contribute to future prevention efforts. Perhaps the most important of these are the provision of antiretroviral therapy (ART) regimens to people living with HIV who have CD4 counts of >500 cells/µl (Test and Treat^^[[1]](#footnote-1)^^), Pre-Exposure Prophylaxis (PrEP) and preventive HIV vaccines. While the first two are potentially actionable in the immediate future, it is recognized that the latter is of hypothetical interest at this time. Equally, a cure may become an important component in the future response to AIDS. However, since this is still in early stages of development, we did not include a cure in the current work. We investigated the potential contribution of each of these other approaches to the long-term reduction in new HIV infections and AIDS mortality. The assumptions for each new technology are shown in Table 3. For each technology we modeled a ‘low’ or pessimistic scenario using conservative assumptions about coverage for each technology and the introduction date for a vaccine, and a ‘high’ or optimistic scenario with higher coverage and earlier availability for vaccines.

*(i)* **Test and Treat (T&T)**

It is well established that ART not only prevents disease progression to AIDS and prolongs survival, but it also reduces infectiousness by reducing HIV viral load^[[2]](#endnote-1),^^[[3]](#endnote-2),^^[[4]](#endnote-3)^ The HPTN 052 trial among serodiscordant couples conclusively showed a 96% reduction in transmission among couples who initiated ART early versus those who waited until the CD4 count of the positive partner dropped below 250 cells/μl.^^[[5]](#endnote-4)^^ Similarly, Donnell and colleagues found a 92% reduction in the transmission of HIV among couples who initiated ART at CD4 counts above 250 cells/μl compared to those who did not.^^[[6]](#endnote-5)^^ A similar protective effect has been reported at the population level in British Columbia, Canada^[[7]](#endnote-6)^, and among injection drug users in the same setting^[[8]](#endnote-7)^. More recently, Tanser and colleagues examined data from a large population-based cohort in rural South Africa and found that the risk of new HIV infection was significantly lower in areas with high ART coverage (>30% of HIV+ population) compared to areas of low coverage (<10%).^^[[9]](#endnote-8)^^ Other studies in population settings published more recently from larger scale population cohorts showed lower effects of ART on HIV transmission, highlighting the importance of high quality programmes ensuring adherence and life-long treatment with highly effective and tolerable regimens to maximize the possible effect of ART on HIV transmission^^[[10]](#endnote-9)^^.

In the ‘Test and Treat’ scenario ART coverage expands even further to include known PLHIV with >500 cells/μl and not part of defined population groups, starting in 2014. Thus the effects of ‘Test and Treat’ in this scenario are those resulting from adding treatment for those with CD4 counts above 500 cells/μl who are not already on ART in the Investment Framework Enhanced scenario. Coverage rates reach 40% and 60% among those with CD4 cell counts > 500 by 2025 under pessimistic and optimistic scenarios respectively, as shown in Table 3. Note the effect of ART in all scenarios is to reduce mortality and infectiousness. We assume that lifetime treatment is required to maintain these benefits. We do not assume that prolonged ART leads to a cure.

We used a cost per patient year of treatment of $515 based on a weighted average median price in 2011 of $145 for first and second line ARVs,^^[[11]](#endnote-10)^^ $222 for average service delivery and monitoring costs^^[[12]](#endnote-11)^^ plus an additional 40% for costs above the facility level for administration, logistics, training, planning etc. The cost per patient treated may decline in the future if treatment can be made even more efficient by reducing visits and lab tests but these reductions could be offset by increases in salary and other health system costs particularly in countries with strong economic growth. Studies of PEPFAR treatment sites have found that costs per patient decline with the maturity of the site. On average per patient costs drop by 31% percent after 18 months (compared to costs at 6 months of operation) and by 42% after 30 months^15^. We estimated the distribution of sites by maturity based on the rate of scale up of the numbers of patient on ART to project that costs per patient would decline to $445 by 2027 and then remain at that level.

*(ii)* **Pre-Exposure Prophylaxis and ARV-based Microbicides**

Pre-Exposure Prophylaxis (PrEP) involves the use of antiretroviral drugs by people who are at risk but not infected with HIV to prevent acquisition of HIV infection. Here we use the term PrEP to refer to all forms of ARV-based prophylaxis including the oral PrEP and vaginal gel formulations already tested in several trials as well as new forms that may become available in the future such as a vaginal ring or an injectable. Several PrEP trials have shown that PrEP can be effective if patients adhere to the intervention. The CAPRISA 004 trial found that 1% tenofovir gel use lowered HIV risk by approximately 39 percent.^^[[13]](#endnote-12)^^ The iPrEX trial showed that once-daily doses of combined tenofovir (TDF) and emtricitibine (FTC) reduced the risk of acquiring HIV by 44% among men who have sex with men.^^[[14]](#endnote-13)^^ The Partners PrEP trial showed a 75% reduction in risk of acquisition for the negative partner in serodiscordant couples for those using TDF-FTC and 67% for those taking TDF alone.^^[[15]](#endnote-14)^^ The TDF2 trial among men and women in Botswana found a 62% reduction in risk among those taking TDF-FTC daily.^^[[16]](#endnote-15)^^ Two other studies, conducted among women at increased risk of acquiring HIV, FEM-PrEP^^[[17]](#endnote-16)^^ (which used a daily oral dose of TDF/FTC) and VOICE^^[[18]](#endnote-17)^^ (which used daily oral TDF or tenofovir 1% tenofovir vaginal gel), found no evidence of effect. In 2012 the US Food and Drug Administration approved Truvada (TDF/FTC) for the prevention of HIV in individuals who are at high risk of HIV.

Further analysis of the results of these studies suggests that adherence to regular dosing is critical to achieve the prevention effect. As a result, additional approaches to support adherence of existing products are being investigated, and researchers are also working on long-acting forms such as vaginal rings and 3-monthly injections that could produce better adherence than the oral or gel forms currently tested.

For modeling purposes, and consistent with the existing RCT data, we assume that PrEP would be targeting populations at high risk of acquiring HIV. Depending on the setting, these groups would include men who have sex with men, female sex workers, serodiscordant couples, and sexually active adolescents in hyper-endemic settings. As shown in Table 3, we assume that PrEP would only be made available for widespread use until technologies and implementation approaches could ensure good overall adherence. For this study, we assumed that such wide spread use may be possible starting in 2018. Before 2018, we have assumed limited use of PrEP for MSM. Target coverage of PrEP is set at the maximum of 60% of included populations. We have assumed that PrEP implemented in this fashion will reduce the rate of HIV acquisition by 90%. Adherence to taking PrEP regimens has been shown to have a major impact on its effectiveness and counseling and close monitoring will always be a critical component in any PrEP application. High effectiveness of PrEP may be achievable in self-selecting populations and with new long lasting or injectable regimens which will be less sensitive to irregular use. Current costs for PrEP have been estimated at about $250-$350^^[[19]](#endnote-18)^^ per person per year but are based on currently available products. We assume that the cost can be reduced to about $95 per person per year as scale-up increases and new forms of PrEP are produced. This includes costs of about $50 for drugs, $30 for service delivery, and $15 for testing.

*(iii)* **HIV Vaccines**

Research on HIV vaccines has been underway since the identification of the virus. Efforts continue on many fronts because of the tremendous benefits an effective vaccine could bring. The first positive efficacy results from a preventive HIV candidate vaccine trial came from the RV 144 trial in Thailand of a phase III prime-boost HIV vaccine.^^[[20]](#endnote-19)^^ That study found a 31% reduction in new infections among those vaccinated. Work is continuing on this and other vaccine candidates. A follow-up program is moving forward with the goal of improving on RV 144’s efficacy and applying it to HIV subtypes in other parts of the world.^^[[21]](#endnote-20)^^ Other vaccine candidates are moving forward in efficacy trials and recent advances in the identification and characterization of broadly neutralizing antibodies against HIV have given clues to improving a vaccine’s ability to prevent acquisition of infection across a majority of existing strains. ^^[[22]](#endnote-21)^^ The recent failure of the HVTN 505 vaccine trial shows how difficult the development process is. An effective HIV vaccine will not be ready for use soon. Furthermore, there is great uncertainty about the timeline, feasibility, level of efficacy and cost for a future vaccine. For the vaccine analysis, we have assumed that a vaccine with at least 60-80% effectiveness would justify widespread implementation. If such a vaccine did become available we assume that it would be available to all uninfected adults in countries with high HIV prevalence and focused on high- risk populations in countries with concentrated epidemics (Table 3). The cost of a future vaccine is unknown but based on comparisons with vaccines against other diseases, we assume that the cost in low income countries will be $20 per person ($5 for each of three doses plus $5 in implementation costs) in the initial years of introduction dropping to $12 per person after 10 years. For middle income countries we have assumed costs of $55 at launch dropping to $35 after 10 years. Since a vaccine may not provide lifetime protection we have assumed that a booster is needed every 3 to 10 years (Table 3) at a cost of $5 (launch) and $3 (after 10 years) in low income countries and $15/$10 in middle income countries.

**Mathematical Modeling Approach**

To examine the long-term effects we projected the 2011 Investment Framework to 2050 under different scenarios describing the scale-up of current and new prevention technologies.

This model estimates the annual number of new HIV infections occurring among adults by sex and risk group (sex workers and clients, men who have sex with men, people who inject drugs, heterosexual couples in stable relationships and heterosexual men and women with casual partners) as a function of behaviors (number of sexual partners, acts per partner, condom use, age at first sexual encounter, needle sharing) and the characteristics of partners (stage of infection, presence of other sexually transmitted infections, male circumcision, use of combination antiretroviral therapy [ART]). The HIV+ population is tracked by CD4 count and mortality is determined by CD4 count category and ART status. New child infections due to mother-to-child transmission are estimated and tracked as they progress to treatment eligibility and death. Parameter values for progression and mortality rates have been published previously^^[[23]](#endnote-22)^,^[[24]](#endnote-23)^^. Data inputs for each country were drawn from national surveys (Demographic and Health Surveys and AIDS Indicator Surveys) and national progress reports (available on the UNAIDS website) and adjusted to match the prevalence trends from national estimates as reported to UNAIDS.^^[[25]](#endnote-24)^^ The results were adjusted for countries that were not explicitly modeled, so as to represent totals for all 139 low- and middle income countries. Box 1 describes how to obtain copies of the model and data files as well as an interactive version of the analysis described in this paper.

## Structure

The Goals model simulates an HIV epidemic in an adult population aged 15-49. The focus is on this population because most new infections occur in this age group and most survey data on sexual behavior refer to this age group. The adult population is disaggregated by sex and risk group. People enter the model at age 15 and are assumed to not be sexually active until they reach the median age at first sex. Then they are allocated to one of the five risk groups, as shown in Figure 1. The categories were chosen because they are epidemiologically important and information on the proportion of population in the three of the categories (stable couples, multiple partners and sex worker and clients) is available from national surveys.

The risk groups are defined by the behaviors specified in each application, so almost any risk structure might be modeled. However, in practice the standard risk groups are those shown in Figure S1. *Stable couples* are men and women reporting a single partner in the last year. The *multiple partner* category refers to those who report more than one partner in the last year but are not in any of the higher risk categories. *Sex workers and clients* refers to female sex workers and male clients. *MSM* refers to men who have sex with men. *IDU* refers to injecting drug users. While any individual may belong to more than one group, people are classified according to their highest risk.

Figure S1. Risk Structure of Goals


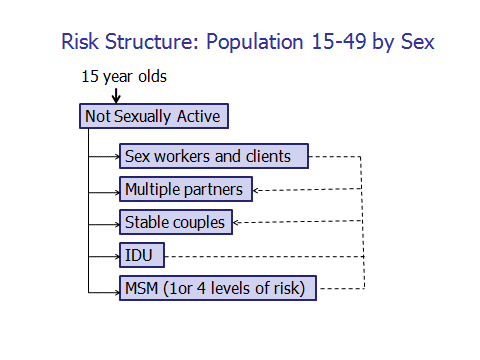


Once a person joins a risk group he or she remains in that risk group until aging out at age 50, dying from non-AIDS cases, dying from AIDS or changing behavior. Behavior change allows people to move from one risk group to a lower risk group. Duration in a risk group may be specified as lifetime or as an average number of years. For example, duration in sex work might be set to 5 or 10 years. Those leaving the highest risk groups (sex work, MSM, IDU) move to the medium risk group (casual sex) and those leaving the casual sex group move to the stable couples risk group.

## Sexual Transmission of HIV

Transmission of HIV from an infected partner to an uninfected partner depends on the characteristics of both partners and the partnership, as shown in Figure S2. For the susceptible partner the important characteristics are the number of partners, if male whether he is circumcised, and whether or not new prevention methods, such as PrEP or vaccines, are used. Transmission from the infected partner is affected by the stage of infection (primary, asymptomatic, or symptomatic), whether the partner is receiving ART and whether the partner has been vaccinated with an HIV vaccine that reduces infectiousness. Characteristics of the partnership that influence transmission are the number of acts per partner per year, whether either partner has a sexually transmitted infection, the type of contact (heterosexual, MSM or needle sharing) and condom use.

Figure S2. Characteristics determining transmission of HIV


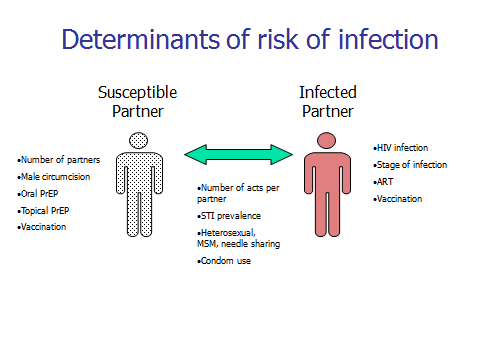


New infections, *I,* are calculated as the susceptible population, X*,* multiplied by the probability of becoming infected, ɩ.

*$I_{s,k,t}=X_{s,k,t}\times\iota_{s,k,t}$*

Where

s = Sex, male or female

k = Risk group

t = Time

The probability of transmission to an uninfected partner during one year, ɩ, is given by the equation below.

Where

P_s’,k,t_ = HIV prevalence in the partner population of risk group k at time *t*

r = Base probability of HIV transmission per act

R_t_ = Multiplier for the effect of stage of infection

C_k,t_ = Multiplier for effect of condom use

MC_k,t_ = Multiplier for effect of male circumcision

S_k,t_ = Multiplier for effect of sexually transmitted infections

Pr_k,t_ = Multiplier for effect of PrEP

V_k,t_ = Multiplier for effect of HIV vaccines

a = Number of acts per partner per year

n = Number of partners per year

Prevalence in the partner population is defined differently for each risk group. For sex workers or clients and those with multiple partners, it is the prevalence of the population of the opposite sex in the same risk group. For MSM it is the prevalence in the MSM group. For stable couples the prevalence in the partner population is a weighted average of the prevalence in each risk group *P* multiplied by the proportion of contacts with that risk group *A*.

$$P_{s,k,t}=\sum_{k} P_{s',k,t}\times A_{s,k,t}$$

For those in stable relationships the proportion of contacts with each risk group is calculated from the number of people in each risk group N_k_ and the proportion married *m*.

$$A_{s,k,t}=\sum_{k} m_{s,k}\times N_{s,k,t}\div\sum_{k} N_{s,k,t}$$

The effect of stage of infection is calculated as a weighted average of infectiousness by stage *i* and the proportion of the population in each stage.

$$R_{t}=\sum_{i} \sum_{k} \sum_{s} Y_{i,k,s,t}\times IM_{i}\div\sum_{i} \sum_{k} \sum_{s} Y_{i,k,s,t}$$

Where

Y_i,k,s,t_ = HIV+ population in stage *i* risk group k sex *s* at time *t*.

IM_i_ = Infectiousness multiplier of stage *i*

The stages are primary infection, asymptomatic, symptomatic (CD4 count < 200 cells/μl) and on ART.

The impact of sexually transmitted infections (STIs) is calculated from the prevalence of STIs in either partner and the increase in transmission when STIs are present.

$$S_{k,t}=1+Sm\times Sp_{k,t}$$

Where

Sm = Multiplier on the probability of transmission when either partner has an STI

Sp_k,t_ = Proportion of risk group k with an STI at time *t*

## Transmission among Injecting Drug Users

The probability of transmission through drug injections is determined by a force of infection variable that expresses the rate of contact between sharing groups, *f*, the prevalence of HIV among IDU *P*, the multiplier for stage of infection *R* and any effects of new technologies such as vaccines *V* or PrEP *Pr*.

$$f_{t}\times P_{k,t}\times R_{t}\times\Pr_{k,t}\times V_{k,t}$$

## Impact of Biomedical Interventions

The impacts of biomedical interventions are included in the transmission equation by the multipliers for condoms, *C*, male circumcision, *MC*, PrEP, *Pr*, and vaccines, *V.* The calculation of each multiplier follows the same form, (1 – effectiveness) multiplied by the percentage of the population covered.

$$C_{k,t}=1-Ce\times Cc_{k,t}$$

Where

Ce = Condom effectiveness

Cc_k,t_ = Proportion of risk group k using condoms at time *t*

Similarly,

$$\begin{matrix} MC_{k,t}=1-MCe\times MCc_{k,t} \\ \Pr_{k,t}=1-\Pr e\times\Pr c_{k,t} \\ V_{k,t}=1-Ve\times Vc_{k,t} \end{matrix}$$

## Impact of Behavior Change Interventions

The impact of behavior change is expressed in the transmission equation by the variables for condom use, *Cc,* and the number of partners, *n*. Behavior change interventions can also affect age at first sex, which determines when people become sexually active, and needle sharing among IDUs, which determined the proportion of injecting drug users that is susceptible to infection.

The impact of interventions on each of these behaviors is determined by an impact matrix that describes the impact of each intervention on each behavior for each risk group. The impact matrix is based on an extensive literature search which is described in detail elsewhere^[[26]](#endnote-25)^.

The impact matrix describes behavior changes for each behavior and each risk group as shown in Tables 1-4 below.

For condoms, Table 1, impact is calculated as a reduction in the non-use of condoms in order to allow for the aggregation of impacts when several interventions are present. Thus, condom use is calculated as one minus the non-use of condoms in the base year multiplied by the product across all interventions of the increase in coverage of each intervention and it impact on condom non-use:

*Cc_t_* = 1 – (1 - *Cc_o_*) x ∏_i_ (Coverage_i,t_ – Coverage_i,0_) x CondomImpact_i_

Table 1. Reduction in Non-Use of Condoms when Exposed to Behavioral Interventions

| **Intervention** | **Reduction in non-use of condoms** | | | |  |
| --- | --- | --- | --- | --- | --- |
|  | **High** | **Medium** | **Low** | **MSM** | **IDU** |
| **Community mobilization** |  | -10 | -2.5 |  |  |
| **Mass media** |  | -11.6 | -17 |  |  |
| **VCT** | -44.2 | -23.4 | -16.1 |  |  |
| **Condoms** | -85.1 | -55.1 | -5.3 |  |  |
| **Youth: in-school** |  | -15.7 |  |  |  |
| **Youth: out-of-school** |  | -16.6 |  |  |  |
| **Workplace programs** |  | -17.1 | -1.0 |  |  |
| **Outreach** | -42.5 | 0 |  | -36.3 | -17.9 |
| **MSM: lubricants** | 0 | 0 |  |  |  |
| **IDU: needle sharing** | -41.5 | 0 |  |  |  |
| **IDU: drug substitution** | 0 | 0 |  |  |  |

The impact on the number of sexual partners (Table 2) is determined as the number of partner in the base year multiplied by the product of the increase in coverage and the impact of each intervention.

*n_t_*  = *n_0_* x ∏_i_ (Coverage_i,t_ – Coverage_i,0_) x Number of Partners Impact_i_

Table 2. Reduction in Number of Partners per Year when Exposed to Behavioral Interventions

|  | **Reduction in # of partners** | | |  |  |
| --- | --- | --- | --- | --- | --- |
|  | **High** | **Medium** | **Low** | **MSM** | **IDU** |
| **Community mobilization** |  |  |  |  |  |
| **Mass media** |  | -4.2 |  |  |  |
| **VCT** |  | -13.1 |  |  |  |
| **Condoms** |  | -13 |  |  |  |
| **Youth: in-school** |  | -18.3 |  |  |  |
| **Youth: out-of-school** |  | -18.3 |  |  |  |
| **Workplace programs** |  | -35.5 |  |  |  |
| **Outreach** | -11.2 |  |  | -10.1 | -1.2 |
| **MSM: lubricants** |  |  |  |  |  |
| **IDU: needle sharing** |  |  |  |  |  |
| **IDU: drug substitution** | -46 |  |  |  |  |

The impact on age at first sex, Table 3, is calculated as age at first sex in the base year multiplied by the product of the increase in coverage and the impact of each intervention.

*AFS_t_* = *AFS_o_* x ∏_i_ (Coverage_i,t_ – Coverage_i,0_) x Age at First Sex Impact_i_

Table 3. Increase in Age at First Sex When Exposed to Behavioral Interventions

| **Community mobilization** | -0.3 |
| --- | --- |
| **Mass media** |  |
| **VCT** |  |
| **Condoms** |  |
| **Youth: in-school** | 0.1 |
| **Youth: out-of-school** | 0.1 |
| **Workplace programs** |  |
| **Outreach** |  |
| **MSM: lubricants** |  |
| **IDU: needle sharing** |  |
| **IDU: drug substitution** |  |

Similarly the impact on needle sharing, Table 4, is calculated as the proportion sharing needles in the base year multiplied by the product of the increase in coverage and the impact of each intervention.

*NS_t_* = NS*_o_* x ∏_i_ (Coverage_i,t_ – Coverage_i,0_) x Needle Sharing Impact_i_

Table 4. Reductions in Unsafe Injecting Behaviors When Exposed to Behavioral Interventions

|  | **Needle Sharing** |
| --- | --- |
| **Outreach** | -17.9 |
| **IDU: needle sharing** | -50.7 |
| **IDU: drug substitution** | -65.0 |

## Progression of HIV-Infected Population

The HIV+ population is tracked by CD4 count. The model structure has seven CD4 compartments as shown in Figure 1b in the main paper. The compartments were selected on the basis of eligibility criteria and mortality patterns.

We assume that most newly infected people start with CD4 counts above 500, although some portion, p, can start at 350-499. The transition probabilities λ1, λ2, λ3, λ4, λ5 and λ6 represent the probability of progressing from one CD4 category to the next. In each category there is some probability of death from HIV-related causes, designated as μ1, μ2, μ3, μ4, μ5, μ6 and μ7 as well as a chance of death from non-AIDS causes, μ0 (not shown in the figure). The probability of HIV-related death increases as CD4 counts decrease.

The number of people in the different CD4 count categories represents the HIV-infected population not on ART. The number of people eligible for treatment is the number in each CD4 count category that is below the recommended level for initiating ART.

Depending on the eligibility criterion and the level of first line ART coverage, a percentage of those eligible for treatment will start first line ART (c1, c2, c3, c4, c5, c6, c7). Those on ART are categorized by their CD4 count at the initiation of treatment. The model does not track the temporal decline of CD4 counts of those on treatment. Those on first line ART have a probability of failure depending on their CD4 count at initiation, α1, α2, α3, α4, α5, α6 and α7.

The number starting ART each year is determined by the assumed coverage and the number of people in the eligible for treatment. We assume that those starting on ART will be distributed among the eligible CD4 categories such that an equal percentage of people in each eligible CD4 category initiate treatment.

## Allocation of New ART Patients by CD4 Count

The number of people on ART in each year is an input to Spectrum. It is used to determine the number of people newly starting ART in each year in order to achieve the specified number of patients. New ART patients need to be allocated across CD4 categories. Information from treatment cohorts on the distribution of new patients by CD4 count is based on a time when almost all countries defined eligibility for treatment as those with CD4 counts under 200. As most countries are now moving to the new WHO guidelines as <350, past patterns may not indicate future patterns.

We have considered two options for allocating new ART patients by CD4 count:

1. Start the same proportion on ART from each eligible CD4 category
2. Allocate new ART patients on the basis of expected AIDS mortality without ART.

When comparing the two methods with data from southern Africa treatment sites it appears that the first method (allocating an equal proportion from all eligible CD4 categories) tends to under-estimate the number starting at low CD4 counts and over-estimate those starting at higher CD4 counts. Weighting the allocations by expected AIDS mortality produced the opposite results, there are too many starting at low CD4 counts and too few at higher CD4 counts. An average of the two methods produces a pattern that matches the data reasonably well. Therefore, Spectrum calculates allocations according to both methods and averages the results.

## Demographic Processes

People enter the model when they reach age 15. The number of people reaching age 15 in each year is provided to Goals from the demographic projection component of Spectrum. Similarly, people age out of the adult population in Goals when they reach the age of 50. Those aging out at 50 are assumed to have the same characteristics as the rest of the adult population in terms of risk group and HIV status.

In addition to aging, adults in Goals are also subject to non-AIDS mortality which is supplied to Goals by the demographic projection model in Spectrum based on age-specific non-AIDS mortality rates.

## Parameter Values

Values of key parameters and the sources are shown in Table 5.

| **Parameter** | **Value** | **Source** |
| --- | --- | --- |
| Transmission of HIV per act (female to male) | 0.0011 | Baggeley *et al^[[27]](#endnote-26)^.*, Gray *et al.* |
| Multiplier on transmission per act for   - Male to female - Presence of STI - MSM contacts | 1.0  8  2.6 | Galvin and Cohen^[[28]](#endnote-27)^, 2.2-11.3  Powers *et a.l^[[29]](#endnote-28)^.* 5.1-8.2  Vittinghoff *et al^[[30]](#endnote-29)^.* |
| Relative infectiousness by stage of infection   - Primary infection - Asymptomatic - Symptomatic - On ART | 9 –  40  1  7  0.04 – 0.08 | Boily *et a.l^[[31]](#endnote-30)^.* 9.17 (4.47-18.81)  Pinkerton^[[32]](#endnote-31)^  Reference stage  Boily *et al^6^.* 7.27 (4.45-11.88)  Cohen *et al.^[[33]](#endnote-32)^*  Attia *et al.^[[34]](#endnote-33)^* |
| Efficacy in reducing HIV transmission   - Condom use - Male circumcision - PrEP - Microbicide | 0.8  0.6  0.55 – 0.73  0.6 | Weller and Davis^[[35]](#endnote-34)^  Auvert *et al^[[36]](#endnote-35)^*, Gray *et al*. (2007)^[[37]](#endnote-36)^, Bailey *et al*.^[[38]](#endnote-37)^  Grant *et al.^[[39]](#endnote-38)^*  Partners PrEP Study  Abdool Karim *et al.^[[40]](#endnote-39)^* |

The time in each CD4 count category is shown in Table 7. These values have been determined by starting with values in the published literature and then fitting the model to data from the ALPHA network. Progression from infection to death is slower for young people than older people. The ALPHA network analysis^1^ found a median time from infection to AIDS death of 12.8 years for those aged 15-24, 10.6 years for 25-34, 7.5 years for 35-44, and 56 years for 45+. They found no difference by sex once survival was adjusted for age. We have used these data to develop parameter sets for these four age groups.

A recent publication by Johansson *et al.* summarizes data from 6 countries on mortality by CD4 count for those not on ART^[[41]](#endnote-40)^. They found annual mortality of 0.8 (0.7-0.9) for those with CD4 counts <50 (from one study in Thailand), 0.355 (0.335-0.375) for those with CD4 counts between 50 and 199 from 6 studies, and 0.109 (0.093-0.125) for those with CD4 counts between 250 and 350 from six studies.

Information on the rate of CD4 cell count decline is available from a several studies, with one providing declines by CD4 category (Table 6).

Table 6. Annual declines in CD4 count among HIV-positive adults

| **Study** | **Annual Rate of Decline in CD4 Count** |
| --- | --- |
| CASCADE, Wolbers et al.^[[42]](#endnote-41)^ | 61 (46-81) Fixed effects  74 (31-145) By patient  114 (32-229) Last 2 tests |
| MACS, Mellors et al. JAMA, 2007^[[43]](#endnote-42)^ | 64 (8-136) |
| CNICS, SFMHS, REACH, Rodriguez et al.^[[44]](#endnote-43)^ | 50 (46-55) |
| Williams et al. JID, 2006^[[45]](#endnote-44)^ | 85 South Africa  65 Zambia |
| Cape Town, Holmes et al.^[[46]](#endnote-45)^ | >500: 47 (40-54)  351-500: 31 (23-38)  201-350: 20 (14-27) |

For this model the parameter values were determined by using the literature values as a starting point and fitting the model to Weibull curves of progression to mortality by age from the ALPHA network. We assumed that the rate of CD4 decline is constant across all categories and that mortality varies by CD4 category but not by age. The resulting parameter values are shown in Table 7.

Table 7. Number of years in each CD4 count category by age and sex

|  | Male | | | | Female | | | |
| --- | --- | --- | --- | --- | --- | --- | --- | --- |
|  | 15 - 24 | 25 - 34 | 35 - 44 | 45 -54 | 15 - 24 | 25 - 34 | 35 - 44 | 45 -54 |
| > 500 | 8.02 | 6.95 | 3.96 | 2.47 | 8.02 | 6.95 | 3.96 | 2.47 |
| 350 - 499 | 3.35 | 2.43 | 1.53 | 0.94 | 3.35 | 2.43 | 1.53 | 0.94 |
| 250 - 349 | 2.23 | 1.62 | 1.02 | 0.63 | 2.23 | 1.62 | 1.02 | 0.63 |
| 200 - 249 | 1.12 | 0.81 | 0.51 | 0.31 | 1.12 | 0.81 | 0.51 | 0.31 |
| 100 - 199 | 2.23 | 1.62 | 1.02 | 0.63 | 2.23 | 1.62 | 1.02 | 0.63 |
| 50 - 99 | 1.12 | 0.81 | 0.51 | 0.31 | 1.12 | 0.81 | 0.51 | 0.31 |

Mortality while on ART has been estimated by the IeDEA (International Epidemiologic Database to Evaluate AIDS) Consortium which has analyzed data from 50,000 patients in East Africa to determine mortality of patients on ART by CD4 count at treatment initiation. In the future, mortality patterns will become available for other regions: southern Africa, West Africa, Latin America and Asia. The actual patterns used in Spectrum vary by age, sex and time on ART: 0-6 months, 7-12 months and more than 12 months as shown in Table 8.

Table 8. Annual mortality (per 100 person-years) on ART by CD4 count, age, sex and time on ART

| **CD4 Count by Time on ART** | **Males** | | | | **Females** | | | |
| --- | --- | --- | --- | --- | --- | --- | --- | --- |
| **0-6 Months** | 15-24 | 25-34 | 35-44 | 45+ | 15-24 | 25-34 | 35-44 | 45+ |
| >500 | 5.84 | 4.42 | 3.91 | 4.40 | 4.22 | 3.19 | 2.82 | 3.18 |
| 350-500 | 10.53 | 7.97 | 7.05 | 7.93 | 7.61 | 5.76 | 5.09 | 5.73 |
| 250-349 | 14.33 | 10.85 | 9.59 | 10.79 | 10.35 | 7.83 | 6.93 | 7.80 |
| 200-249 | 11.13 | 8.43 | 7.45 | 8.38 | 8.04 | 6.09 | 5.38 | 6.06 |
| 100-199 | 12.78 | 9.68 | 8.55 | 9.63 | 9.23 | 6.99 | 6.18 | 6.95 |
| 50-99 | 22.74 | 17.21 | 15.22 | 17.13 | 16.42 | 12.43 | 10.99 | 12.37 |
| <50 | 41.69 | 31.56 | 27.90 | 31.40 | 30.11 | 22.79 | 20.15 | 22.68 |
| **7-12 months** |  |  |  |  |  |  |  |  |
| >500 | 1.76 | 1.17 | 1.19 | 1.42 | 1.17 | 0.78 | 0.80 | 0.95 |
| 350-500 | 2.36 | 1.57 | 1.60 | 1.91 | 1.58 | 1.05 | 1.07 | 1.28 |
| 250-349 | 2.16 | 1.44 | 1.47 | 1.75 | 1.44 | 0.96 | 0.98 | 1.17 |
| 200-249 | 1.72 | 1.14 | 1.16 | 1.39 | 1.15 | 0.76 | 0.78 | 0.93 |
| 100-199 | 2.11 | 1.40 | 1.43 | 1.71 | 1.41 | 0.94 | 0.96 | 1.14 |
| 50-99 | 2.70 | 1.80 | 1.83 | 2.19 | 1.81 | 1.20 | 1.23 | 1.46 |
| <50 | 3.43 | 2.28 | 2.33 | 2.78 | 2.29 | 1.52 | 1.56 | 1.86 |
| **>12 months** |  |  |  |  |  |  |  |  |
| >500 | 1.08 | 0.72 | 0.74 | 0.88 | 0.73 | 0.48 | 0.49 | 0.59 |
| 350-500 | 1.45 | 0.97 | 0.99 | 1.18 | 0.97 | 0.65 | 0.66 | 0.79 |
| 250-349 | 1.33 | 0.89 | 0.90 | 1.08 | 0.89 | 0.59 | 0.60 | 0.72 |
| 200-249 | 1.06 | 0.70 | 0.72 | 0.86 | 0.71 | 0.47 | 0.48 | 0.57 |
| 100-199 | 1.30 | 0.87 | 0.88 | 1.05 | 0.87 | 0.58 | 0.59 | 0.71 |
| 50-99 | 1.67 | 1.11 | 1.13 | 1.35 | 1.11 | 0.74 | 0.76 | 0.90 |
| <50 | 2.12 | 1.41 | 1.44 | 1.71 | 1.42 | 0.94 | 0.96 | 1.15 |

1. While the term ‘Test and Treat’ has been used in very different ways by different experts and communities, for this paper we use the term to mean the provision of ART to people living with HIV who are not eligible under the new 2013 WHO treatment guidelines (with CD4>500 cells//µl and not part of a defined population group). [↑](#footnote-ref-1)
2. Montaner J. Treatment as prevention—a double hat-trick. The Lancet. Vol 378 July 16, 2011. Pages 208-209 [↑](#endnote-ref-1)
3. Nosyk B, Montaner JSG (2012) The Evolving Landscape of the Economics of HIV Treatment and

   Prevention. PLoS Med 9(2): e1001174. doi:10.1371/journal.pmed.1001174 [↑](#endnote-ref-2)
4. Nosyk B, Audoin B, Beyrer C, Cahn P, Granich R, Havlir D, Katabira E, Lange C, Lima VD, Patterson T, Strathdee S, Williams B, Montaner JSG. Examining the evidence on the causal effect of highly active antiretroviral therapy on transmission of human immunodeficiency virus using the Bradford Hill criteria. IN PRESS, AIDS. [↑](#endnote-ref-3)
5. Cohen MS, Chen YQ, McCauley M, et al. (August 2011). Prevention of HIV-1 infection with early antiretroviral therapy. N. Engl. J. Med. 365 (6): 493–505. doi:10.1056/NEJMoa1105243. PMC 3200068. PMID 21767103 [↑](#endnote-ref-4)
6. 11 Donnell D, Baeten JM, Kiarie J, Thomas KK, Stevens W, Cohen CR, et al. Heterosexual HIV-1 transmission after initiation of antiretroviral therapy: a prospective cohort analysis (12 June 2010) The Lancet 375(9731):2092-2098. [↑](#endnote-ref-5)
7. Montaner JS, Lima VD, Barrios R, Yip B, Wood E, Kerr T, Shannon K, Harrigan PR, Hogg RS, Daly P, Kendall P. Association of highly active antiretroviral therapy coverage, population viral load, and yearly new HIV diagnoses in British Columbia, Canada: a population-based study. Lancet 2010;376(9740):532-9. [↑](#endnote-ref-6)
8. Wood E, Kerr T, Marshall B, Li K, Zhang R, Hogg RS, Harrigan PR, Montaner JSG. Longitudinal community plasma HIV-1 RNA concentrations and incidence of HIV-1 among injecting drug users: prospective cohort study. BMJ 2009;338:b1649. [↑](#endnote-ref-7)
9. Tanser F, Barnighausen T, Graspa E, Zaidi J, Newell ML (2013) High coverage of ART associated with decline in risk of HIV acquisition in rural Kwa-Zulu Natal, South Africa Science 339:966. doi:10.1126/science.1228160. [↑](#endnote-ref-8)
10. Jia Z, Ruan Y, Li Q, Xie P, Li P, Wang X et al. Antiretroviral therapy to prevent HIV transmission in serodiscordant couples in China (2003-2011): a national observational cohort study. The Lancet, Published online December 1, 2012 nhttp://dx.doi.org/10.1016/S0140—6736(12)61898-4. [↑](#endnote-ref-9)
11. World Health Organization. Global HIV/AIDS response: epidemic update and health sector progress towards universal access: progress report 2011, WHO, UNAIDS, UNICEF. [↑](#endnote-ref-10)
12. Menzies NA, Berruti AA, Blandford JM. The Determinants of HIV Treatment Costs in Resource Limited Settings PLoS ONE 7(11): e48726.doi:10.1371/journal.pone.0048726 [↑](#endnote-ref-11)
13. Abdool Karim, Q et al. on behalf of the CAPRISA 004 Trial Group Effectiveness and Safety of Tenofovir Gel, an Antiretroviral Microbicide, for the Prevention of HIV Infection in Women Science 2010; Vol. 329 no. 5996 pp. 1168-1174 DOI: 10.1126/science.1193748 [↑](#endnote-ref-12)
14. Grant RM, Lama JR, Anderson PL, McMahan V, Liu AY, Vargas L et al. (2010) Preexposure Chemoprophylaxis for HIV Prevention in Men Who Have Sex with Men N Engl J Med 363(27):2587-2599. [↑](#endnote-ref-13)
15. Baeten JM, Donnell D, Ndase P, Mugo N, Campbell JD, Wangisi J et al. (2012) Antiretroviral Prophylaxis for HIV Prevention in Heterosexual Men and Women N Engl J Med 367(5):399-410. [↑](#endnote-ref-14)
16. Thigpen MC, Kebaabetswe PM, Paxton LA, Smith DK, Rose CE, Segolodi TM et al. (2012) Antiretroviral Preexposure Prophylaxis for Heterosexual HIV Transmission in Botswana N Engl J Med 2012;367:423-34.

    DOI: 10.1056/NEJMoa1110711. [↑](#endnote-ref-15)
17. Van Damme L et al. The FEM-PrEP Trial of Emtricitabine/Tenofovir Disoproxil Fumarate (Truvada) among African Women. 19th Conference on Retroviruses and Opportunistic Infections, Seattle, abstract 32LB, 2012. [↑](#endnote-ref-16)
18. Anticipating the Results of VOICE | Microbicide Trials Network [Internet]. [cited 24th Jan 2013]. Available at: http://www.mtnstopshiv.org/node/4667. [↑](#endnote-ref-17)
19. Bill & Melinda Gates foundation. Oral PrEP in South Africa. Bottom-up cost model. Spreadsheet available at: http://www.gatesfoundation.org/grantseeker/Documents/program-cost-model-rsa.xls; 2011. [↑](#endnote-ref-18)
20. Rerks-Ngarm, S.; Pitisuttithum, P.; Nitayaphan, S.; Kaewkungwal, J.; Chiu, J.; Paris, R.; Premsri, N.; Namwat, C. et al. (2009). "Vaccination with ALVAC and AIDSVAX to Prevent HIV-1 Infection in Thailand". New England Journal of Medicine 361 (23): 2209–2220. doi:10.1056/NEJMoa0908492 [↑](#endnote-ref-19)
21. Sanofi-Pasteur. “HIV Vaccines: Building on Success - RV144 Follow-Up Studies” <http://www.sanofipasteur.com/sp-media/SP_CORP4/EN/161/2175/ANNEXE%201%20-%20P5%20Factsheet_FINAL.pdf> Accessed January 17, 2013. [↑](#endnote-ref-20)
22. Burton DR, et al. “Broadly neutralizing antibodies present new prospects to counter highly antigenically diverse viruses.” *Science*. 2012 Jul 13;337(6091):183-6. [↑](#endnote-ref-21)
23. Stover J, Brown T, Marston M. Updates to the Spectrum/Estimation and Projection Package (EPP) model to estimate HIV trends for adults and children (2012) Sex Trans Infect 2012;88:i11-ii16. doi:10.1136/sextrans-2012-050640. [↑](#endnote-ref-22)
24. Yiannoutsos CT, Johnson LF, Boulle A, Musick BS, Gsponer T, Balestre E, et al. Estimated mortality of adult HIV-infected patients starting treatment with combination antiretroviral therapy Sex Transm Infect 2012;88i33-i43. doi:10.1136/sextrans-2012-050658. [↑](#endnote-ref-23)
25. Available through AIDSInfo on the UNAIDS website at http://www.unaids.org/en/dataanalysis/datatools/aidsinfo/ [↑](#endnote-ref-24)
26. Bollinger LA, How can we calculate the “E” in “CEA” *AIDS* 2008, 22(suppl 1): S51-S57. [↑](#endnote-ref-25)
27. Baggaley RF, Fraser C. Modelling sexual transmission of HIV: testing the assumptions, validating the predictions. Curr Opin HIV AIDS. 2010; **5**(4): 269-76. [↑](#endnote-ref-26)
28. Galvin and Cohen, "The Role of Sexually Transmitted Diseases in HIV Transmission" Nature Reviews Microbiology Volume 3, January 2004, pps. 33-42. [↑](#endnote-ref-27)
29. Powers KA, Poole C, Pettifor AE, Cohen MS Rethinking the heterosexual infectivity of HIV-1: a systematic review and meta-analysis The Lancet Published on line August 5, 2008 DOI:10.1016/S1273-3099(08)70156-7. [↑](#endnote-ref-28)
30. Vittinghoff E, Douglas J, Judson F, McKirnan D, MacQueen K, Buchbinder SP. Per-Contact Risk of Human Immunodeficiency Virus Transmission between Male Sexual Partners Am J Epidemiol (1999)150:3;306-31 suggests 0.0016/0.0011. [↑](#endnote-ref-29)
31. Boily MC, Baggaley RF, Wang L, Masse B, White RG, Hayes RJ, Alary M. Heterosexual risk of HIV-1 infection per sexual act: systematic review and meta-analysis of observational studies *Lancet Infect Dis* 2009; 9: 118-29. [↑](#endnote-ref-30)
32. Pinkerton SD. Probability of HIV transmission during acute infection in Rakai, Uganda. AIDS Behav. 2008; **12**(5): 677-84. [↑](#endnote-ref-31)
33. Cohen MS, Chen YQ, McCauley M, Gamble T, Hosseinipour MC, Kumarasamy N, *et al.* Prevention of HIV-1 Infection with Early Antriretroviral Therapy N Engl J Med 2011; 10.1056/NEJMoa1105243. [↑](#endnote-ref-32)
34. Attia S, Egger M, Muller M, Zwahlen M, Low N. Sexual transmission of HIV according to viral load and antiretroviral therapy: systematic review and meta-analysis. AIDS 2009, 23:1-8. [↑](#endnote-ref-33)
35. Weller S, Davis, K. Condom effectiveness in reducing heterosexual HIV transmission (Cochrane Review). In: The Cochrane Library, Issue 1, 2004. Chichester, UK: John Wiley & Sons, Ltd. [↑](#endnote-ref-34)
36. Auvert B, Puren A, Taljaard D, Lagarde E, JoëlleTambekou-Sobngwi, RémiSitta. The impact of male circumcision on the female-to-male transmission of HIV : Results of the intervention trial : ANRS 1265. IAS 2005: INSERM, France; 2005. [↑](#endnote-ref-35)
37. Bailey RC, Moses S, Parker CB, Agot K, Maclean I, Krieger JN, et al. Male circumcision for HIV prevention in young men in Kisumu, Kenya: a randomised controlled trial. Lancet. 2007; **369**(9562): 643-56. [↑](#endnote-ref-36)
38. Bailey RC, Moses S, Parker CB, Agot K, Maclean I, Krieger JN, et al. Male circumcision for HIV prevention in young men in Kisumu, Kenya: a randomised controlled trial. Lancet. 2007; 369(9562): 643-56. [↑](#endnote-ref-37)
39. Grant RM, Lama JR, Anderson PL, McMahan V, Liu AY, Vargas L. Preexposure Chemoprophylaxis for HIV Prevention in Men Who Have Sex with Men New Engl J Med 2010, 10.1056/NEJMoa1011205. [↑](#endnote-ref-38)
40. Karim QA, Karim SSA, Frohlich J, Grobler AC, Baxter C, Mansoor LE, *et al.* Effectiveness and Safety of Tenofovir Gel, an Antoretroviral Microbicide, for the Prevention of HIV Infection in Women. *Science* 329; 1168-1174 (September 2010). [↑](#endnote-ref-39)
41. Johansson KA, Robberstad B, Norheim OF. Further benefits by early start of HIV treatment in low income countries: survival estimates of early versus deferred antiretroviral therapy. AIDS Research and Therapy 2010, 7:3. [↑](#endnote-ref-40)
42. Wolbers M, Babiker A, Sabin C, Young J, Dorrucci M, Chene G, et al. Pretreatment CD4 cell slope and progression to AIDS or death in HIV-infected patients initiating antiretroviral therapy--the CASCADE collaboration: a collaboration of 23 cohort studies. PLoS Med. 2010; 7(2): e1000239. [↑](#endnote-ref-41)
43. Mellors JW, Margolick JB, Phair JP, Rinaldo CR, Detels R, Jacobson LP, et al. Prognostic value of HIV-1 RNA, CD4 cell count, and CD4 Cell count slope for progression to AIDS and death in untreated HIV-1 infection. JAMA. 2007; 297(21): 2349-50. [↑](#endnote-ref-42)
44. Rodriguez B, Sethi AK, Cheruvu VK, Mackay W, Bosch RJ, Kitahata M, et al. Predictive value of plasma HIV RNA level on rate of CD4 T-cell decline in untreated HIV infection. JAMA. 2006; 296(12): 1498-506. [↑](#endnote-ref-43)
45. Williams BG, Korenromp EL, Gouws E, Schmid GP, Auvert B, Dye C. HIV infection, antiretroviral therapy, and CD4+ cell count distributions in African populations. J Infect Dis. 2006; 194(10): 1450-8. [↑](#endnote-ref-44)
46. Holmes CB, Wood R, Badri M, Zilber S, Wang B, Maartens G, et al. CD4 Decline and Incidence of Opportunistic Infections in Cape Town, South Africa: Implications for Prophylaxis and Treatment. JAIDS Journal of Acquired Immune Deficiency Syndromes. 2006; 42(4): 464-9 10.1097/01.qai.0000225729.79610.b7. [↑](#endnote-ref-45)
